# Improved Efficacy of Delayed Treatment with Human Bone Marrow-Derived Stromal Cells Evaluated in Rats with Spinal Cord Injury

**DOI:** 10.3390/ijms25031548

**Published:** 2024-01-26

**Authors:** Marta Aguado-Garrido, Concepción García-Rama, Lorenzo Romero-Ramírez, Vinnitsa Buzoianu-Anguiano, Enrique Pérez-Rizo, Boris W. Kramer, Jörg Mey

**Affiliations:** 1 Hospital Nacional de Parapléjicos, 45071 Toledo, Spain; 2 Neuroplast BV, 6222 NK Maastricht, The Netherlands; 3 EURON Graduate School of Neuroscience, Maastricht University, 6229 ER Maastricht, The Netherlands

**Keywords:** spinal cord injury, bone marrow, stromal cells, neuroinflammation, cytoprotection

## Abstract

The treatment of spinal cord injury (SCI) with uncultivated human bone marrow-derived stromal cells (bmSCs) prepared by negative selection has been proposed to be therapeutically superior to treatment with stem cells that were expanded in vitro. To explore their use in clinical trials, we studied the functional effects of delayed application at 7 days after SCI by testing different doses of bmSCs. Spinal cord contusion injury was induced in adult male Wistar rats at the thoracic level T9. Human bmSCs were prepared by negative selection without expansion in vitro (NeuroCells^TM^). Treatment consisted of one 150 µL injection into the *cisterna magna* containing 0.5 or 2.5 million fresh bmSCs or 2.5 million bmSCs. The recovery of motor functions was evaluated during a surveillance period of six weeks (6 W), during which spinal cords were assessed histologically. Treatment resulted in a significant, dose-dependent therapeutic effect on the recovery of motor performance. The histological analysis revealed a lower degree of axonal degeneration and better survival of neurons and oligodendrocytes in bmSCs treated rats. Our results support delayed intrathecal application of bmSCs prepared by negative selection without expansion in vitro as a treatment of SCI.

## 1. Introduction

Despite important improvements in rehabilitation, there is no curative treatment for spinal cord injury (SCI) [1]. The use of bone marrow-derived stem cells is currently one of the most promising experimental treatments for SCI [2,3,4]. While the application of neural stem cells has the objective of replacing lost neurons and glia cells [1,5,6,7], the rationale of using mesenchymal stem cells consists mainly in modifying the endogenous response in the tissue, primarily by reducing neuroinflammation [4,8]. Paracrine factors and extracellular vesicles that are released from mesenchymal stem cells are expected to prevent secondary degeneration, reduce scar formation, and support regenerative plasticity after SCI [9]. An accessible source of this type of cell is the bone marrow, which contains hematopoietic and mesenchymal stem cells. Bone marrow-derived stromal cells (bmSCs) pose no risk of tumor formation, which remains an issue with induced pluripotent stem cells [1,10]. The company Neuroplast BV developed a procedure to prepare human bmSCs by a negative selection procedure without cell expansion in culture (patent WO2015/059300A1). After extraction from the iliac crest, depletion of erythrocytes and lymphocytes, followed by characterization with flow cytometry and an in vitro potency assay, these cells (NeuroCells^TM^) are intended for the therapy of neurological pathologies, including SCI and frontotemporal dementia. The safety and efficacy of these cells had to be tested in animal experiments. In a previous study using a rat model of SCI, we demonstrated that animals without additional immune suppression showed no negative response to the injection of human bmSCs. One intrathecal injection of two million NeuroCells at 1–2 h after SCI reduced inflammation and axonal degeneration, and had beneficial effects on motor recovery when compared to a control treatment with methylprednisolone [11]. However, the functional improvements were small and not even significant in comparison with a different control group that received no treatment at all. With the intention of improving survival of the injected bmSCs in the acute phase after SCI, the treatment was later combined with anti-inflammatory application of tauroursodesoxycholic acid, but this failed to produce additional benefits [12]. Thus, we reasoned that cellular treatment would be more beneficial if given when the initial inflammatory peak has passed. A clinical trial with NeuroCells is currently in progress (EudraCT 2018-000805-22), and in this case patients receive cellular treatment six to eight weeks after SCI. An additional issue of these and other related studies is that bmSCs were stored frozen and reconstituted, while a major efficacy is only claimed for fresh cells. The dose of implanted stem cells is also potentially important [3].

The objective of the present study was to assess the safety and therapeutic benefits of a delayed intrathecal injection of bmSCs prepared by negative selection. For this we chose the contusion model of rat SCI at the thoracic level, which is the best available compromise between the demand to mimic clinical cases, practical applicability, and animal welfare (in comparison with contusion at cervical levels and the use of larger animals). Our principal focus was on detecting a meaningful functional benefit of the approach in the chronic phase after treatment. For this purpose, standard sensory–motor tests, such as sensitivity to tactile and heat stimulation, open field evaluation, and motor performance on a rotating rod were complemented with quantitative video analysis. The secondary objective was to evaluate the long-term cellular effects of bmSC treatment, such as neuronal survival and microglial and astrocyte activation.

## 2. Results

**Figure 1:**
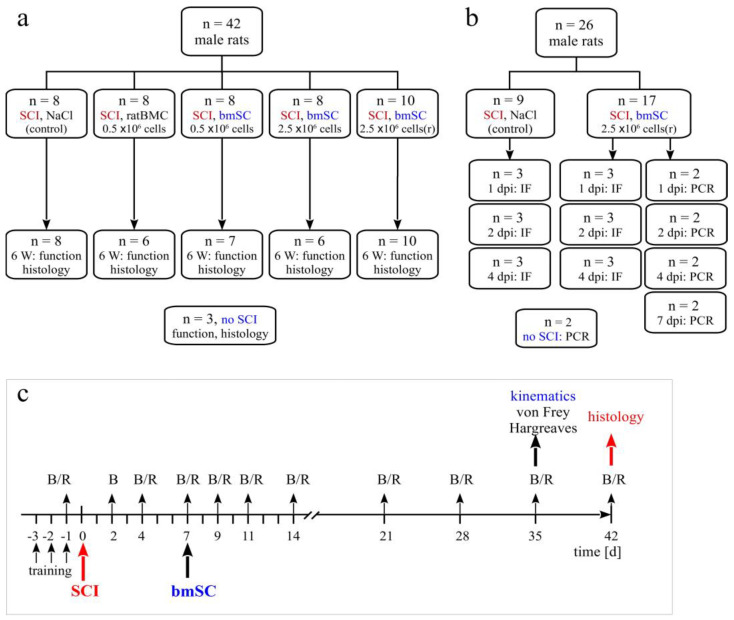
**Study design.** (**a**) For the principal study, 42 male rats were randomly divided into five groups that all received SCI and subsequently one intrathecal injection at 7 dpo. Treatments with human cells were compared with two control groups, which either received an injection of saline (SCI-control) or 500,000 stromal cells prepared from rat bone marrow (SCI-ratBMC). Two doses of fresh NeuroCells, 500,000 cells (SCI-bmSC-05) and 2.5 million cells (SCI-bmSC-25) were compared with NeuroCells that were stored in liquid nitrogen and reconstituted (SCI-bmSC-25r). Animals that appeared to have incomplete lesions (BBB ≥ 1 at 2 dpo) or showed no spontaneous motor recovery (BBB = 0 at 7 dpo before treatment) were excluded from the study. Two animals had to be euthanized because of complications during the intrathecal injection. Three animals received laminectomy to serve as controls for histology. (**b**) In order to detect injected bmSCs in the spinal cord, additional rats were operated and sacrificed at 1, 2, 4, or 7 days after cell injection. Their spinal cord tissues were processed for histology or PCR. Two non-operated animals were used as controls in this experiment. (**c**) The timeline illustrates the treatment and evaluation scheme of the main experiment. On the indicated days, motor functions were assessed in the open field (B) and the RotaRod test (R), additional kinematic and sensory analysis was performed at 5 W after SCI, and rats were sacrificed and perfused for histology at 6 W.

In this study, 42 rats received a severe spinal cord contusion injury at thoracic level T9. One week after SCI, animals were treated with intrathecal injection of human bmSCs in different doses or saline as a control (Figure 1).

### 2.1. General Health Status, Body Weight, and Neuropathic Pain

Intrathecal injections did not affect the general health of the rats, and no adverse effects such as sickness behavior or urinary infections were observed.

#### 2.1.1. Changes in Body Weight

**Figure 2:**
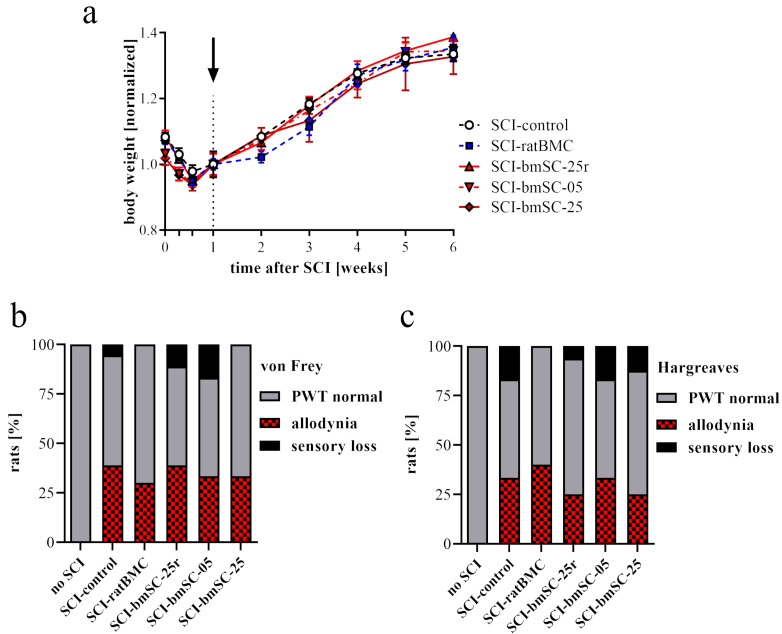
**Body weight and sensory response.** The experimental groups, which received SCI and intrathecal injection at 7 dpo, are abbreviated as follows. SCI-control: saline injection; SCI-ratBMC: 500,000 rat bone marrow-derived stromal cells; SCI-bmSC-25r: 2.5 million live NeuroCells stored in liquid nitrogen and reconstituted; SCI-bmSC-05: 500,000 fresh NeuroCells; SCI-bmSC-25: 2.5 million fresh NeuroCells. (**a**) Following SCI, animals suffered weight loss during the first 4 dpo and subsequently recovered. The surgical intervention at 7 dpo, indicated with a dotted line and arrow, did not cause significant differences in the rate of recovery between different treatment groups, although rats that received rat BMC appeared not to gain body weight during the first days after intervention. (**b**) Sensory response to tactile stimulation was measured with an automated von Frey test. The color code of the columns indicates the percentage of rats that had a PWT of >2 SD below the mean of control animals without SCI (allodynia), a PWT of >2 SD above the mean of control animals (sensory loss), or that were within the range considered normal. (**c**) Sensory response to heat stimulation was determined using an automated Hargreaves test device. Data are presented in the same way as in (**b**).

During the first four days following SCI surgery, the animals lost 8–11% of their body weight, which they recovered with a weight gain of 6.9% in the second week (7–14 dpo), and subsequently 8.5% (W2-3), 9.1% (W3-4), 4.9% (W4-5), and 1.8% (W5-6). There were no significant differences in weight loss or gain between the SCI treatment groups (Figure 2a). However, in a previous study with identical SCI but no second surgery at 7 dpo, the weight gain during the second week was higher (9.5%), indicating that the intervention per se, consisting of ketamine anesthesia, fixation in a stereotactic frame, and insertion of a catheter into the cisterna magna, reduced the initial recovery of body weight.

#### 2.1.2. Effect of bmSC Treatment on Allodynia/Hyperalgesia

The response to tactile stimulation (automated von Frey test) and temperature (Hargreaves, infrared heat) was evaluated at 5 W. Readout in these tests is the paw withdrawal threshold (PWT), and since at this time all animals were capable of withdrawing their hind paws, the validity of PWT for sensory functions was not affected by differences in motor recovery. A normal response was defined as the PWT within two standard deviations from the mean of rats without SCI. A lower response threshold may be considered a sign of allodynia, and a higher threshold as indicative of sensory loss. In both assays, data from all animals without lesion fell within 2 SD around the group mean. In all SCI groups, we observed rats with allodynia and/or sensory loss (Figure 2b,c). Different treatment regimens did not significantly affect the paw withdrawal thresholds [ANOVA, von Frey: F (5, 78) = 1.0, n.s.; Hargreaves: F (5, 66) = 1.6, n.s.], but the percentage of rats that showed reduced PWT to the stimuli was slightly lower in some bmSC treatment groups.

### 2.2. Effect of bmSC Treatment on the Recovery of Sensory–Motor Functions

#### 2.2.1. Evaluation of Motor Recovery in the Open Field

**Figure 3:**
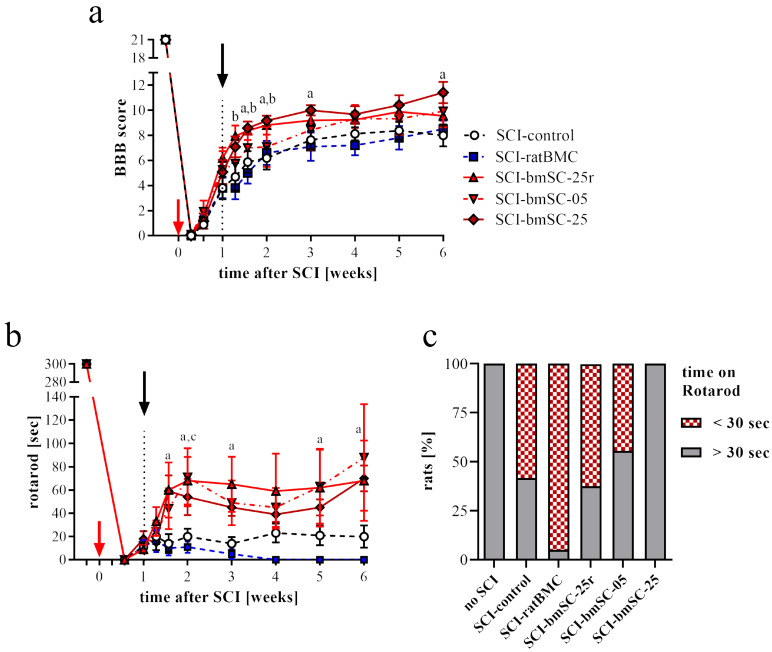
**Recovery of motor functions after SCI.** (**a**) Evaluation in the open field: Mean BBB scores were monitored at 1, 2, 4, 7, 9, 11, 14 dpo and subsequently once per week. Effects of time after treatment and mode of treatment were significant, with the high dose of human bmSCs being therapeutically effective (statistical analysis in main text). In the graph, significant differences (*p* < 0.05) from the SCI-control group at different time points are indicated with letters a (SCI-bmSC-25) and b (SCI-bmSC-25r). Red and black arrows indicate days of SCI and bmSC implantation, respectively. (**b**) RotaRod evaluation, plotted as time [seconds] that animals were able to maintain themselves on the accelerating rod, also revealed some functional recovery with time and a significant effect of bmSC treatment. Significant differences (*p* < 0.05) with the SCI-control group are indicated with letters a (SCI-bmSC-25) and c (SCI-bmSC-05). (**c**) Percentage of animals in each group that maintained their balance on the rotating rod for more than 30 s at the end of the study. Experimental groups are abbreviated as explained for Figure 2.

The standard procedure for the evaluation of motor function after SCI is observation in the open field using the BBB scale (Figure 3a). Using this method at 2 dpo, we ascertained that a bilateral spinal cord contusion was correctly applied (indicated by no movement of the hind legs, i.e., BBB < 1 at 2 dpo). At 7 dpo, just before experimental treatment, the majority of rats were able to move their hind legs. However, three rats that did not regain any mobility at this time were excluded from the evaluation. During the first three weeks after SCI, all rats recovered some sensory–motor performance. Subsequently, no or little additional improvement occurred in the chronic phase (Figure 3a). The statistical analysis showed time after treatment and mode of treatment to be significant without interaction between the two factors [ANOVA, time: F (10, 319) = 228, *p* < 0.0001; treatment: F (4, 319) = 13.8, *p* < 0.0001; interaction: F (40, 319) = 0.74, n.s.]. Compared to saline treatment, post hoc Dunnett’s test demonstrated a highly significant effect of the high doses of human bmSCs (fresh or reconstituted, *p* < 0.001 in both cases) and a significant effect of the low dose of human bmSCs (*p* < 0.05), but no differences between animals treated with ratBMC and the control group (*p* > 0.1). In the chronic phase, only the most effective treatment, intrathecal injection of 2.5 million fresh NeuroCells, resulted in a significantly higher motor score than observed in the SCI-control or SCI-ratBMC groups (ΔBBB = 3.4, *p* < 0.05). Thus, treatment with the higher dose of human bmSCs had a sustained therapeutic benefit, but not the lower dose nor our preparation of stromal cells from rat bone marrow.

#### 2.2.2. Evaluation of Motor Recovery with the RotaRod Test

An independent quantitative assessment of stepping ability was made by means of the RotaRod test, which measures a rat’s ability to maintain equilibrium on a rotating bar. Before SCI, all rats had been trained to perform this task, and at 4 dpo none of the animals that met the BBB inclusion criterion were able to do so. Spontaneous recovery caused a significant increase in the RotaRod score during the first two weeks after SCI in all animals (Figure 3b). With respect to the group average, no further gains occurred subsequently, yet the variability within groups increased dramatically because some animals continued to improve in the task, while others did not try to maintain their position on the rotating rod. Analysis of variance showed time and mode of treatment to be significant without interaction between the two factors [time: F (9, 310) = 122, *p* < 0.0001; treatment: F (4, 310) = 9.1, *p* < 0.0001; interaction: F (36, 310) = 0.73, n.s.]. Compared to saline treatment, post hoc Dunnett’s test demonstrated significant effects of all treatments with human bmSCs (SCI-bmSC-05: *p* < 0.01, SCI-bmSC-25: *p* < 0.05, SCI-bmSC-25r: *p* < 0.001) and no improvement with SCI-ratBMC (*p* > 0.1). The percentage of rats in each experimental group that performed the task for more than 30 s provides a meaningful indicator of their ability to perform weight-supported plantar steps. In the chronic phase (3–6 W), this was achieved by all rats treated with the high dose of fresh NeuroCells but in less than half of those without treatment or with injection of ratBMC (Figure 3c). As in the open field assessment, a therapeutic effect of bmSCs injections was statistically significant in the RotaRod assay.

#### 2.2.3. Evaluation of Motor Recovery Using Kinematic Analysis

**Figure 4:**
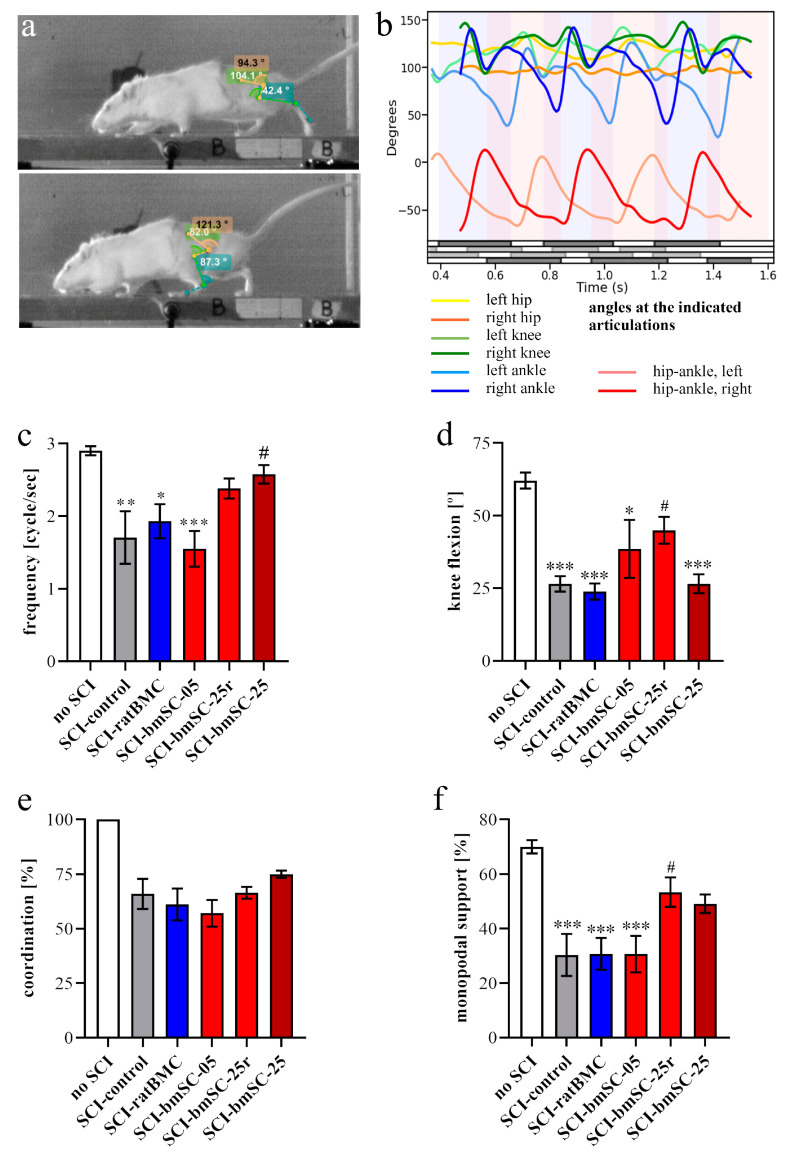
**Kinematics analysis of motor functions.** (**a**) Video frames showing angle measurements at the hip, knee, and ankle during a rat’s movement along a passageway. (**b**) Diagram with measurements used in the evaluation at 5W: range of flexion at the indicated articulations, hip aperture (see Appendix A Figure A1), exact timing of surface contacts of the four limbs (gray lines: left forepaw, left hindpaw, right hindpaw, right forepaw). Quantitative evaluation of video data (for ANOVA, post hoc tests see main text): (**c**) SCI reduced the stepping frequency by half, but much less in rats treated with higher doses of human bmSCs. Compared to SCI-control, the treatment effect became significant with the high dose of fresh bmSCs. (**d**) Limb movement at the knee joints was significantly reduced after SCI. Treatment with the high dose of reconstituted bmSCs improved this parameter. (**e**) Coordination between fore- and hindlimbs was low at 5 W after SCI. None of the cellular treatments led to a significant improvement compared to SCI-control. (**f**) Time of monopodal support while walking was strongly reduced after SCI and partly recovered after treatment with the higher doses of bmSCs. The experimental groups are the same as explained for Figure 2, *, #: *p* < 0.05, **: *p* < 0.01, and ***: *p* < 0.001.

Kinematic video analysis of the rats’ movement was performed at 5 W after SCI (i.e., 4 W after treatment) to validate the open field evaluation and provide additional data on movement coordination (Figure 4a,b). In trained rats who are motivated to walk, stepping frequency is an indicator of motor function. It was dramatically lower in SCI-control animals at 5 W than in rats that had no SCI (Figure 4c). In animals with SCI, treatment significantly affected this parameter [ANOVA, F (4, 57) = 2.95, *p* < 0.05]. Compared to SCI-control, the effect became significant with the highest dose of fresh bmSCs (*p* < 0.05). This was also the only group with a high proportion of rats (>75%) that performed well in the RotaRod task (Figure 3c), an independent indicator of stepping ability.

**Figure A1:**
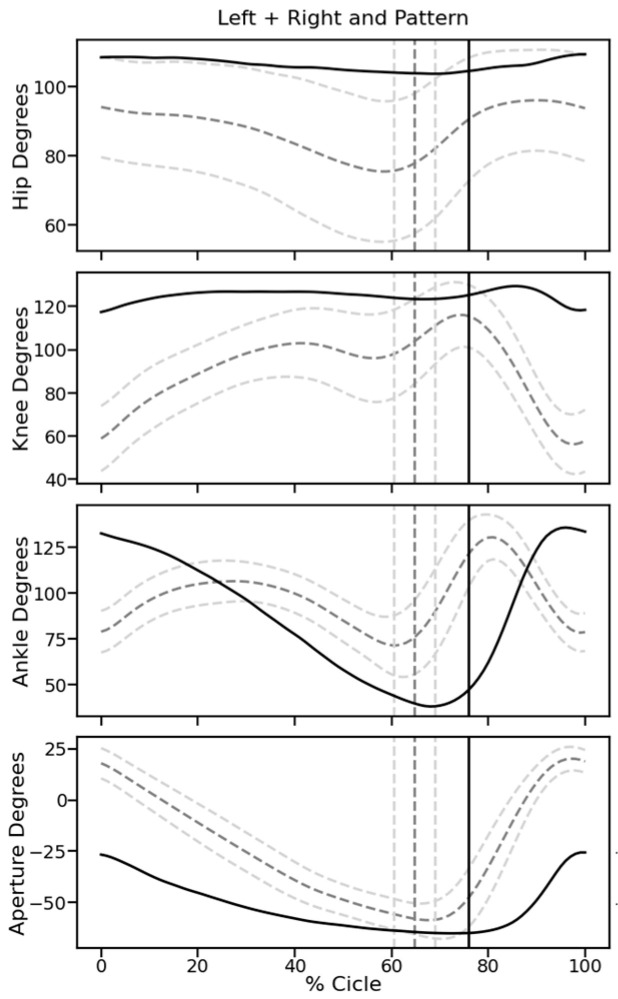
**Kinematics analysis of locomotor functions.** Plots represent the readouts of the flexion-extension angles of the hip, ankle, knee, and aperture angle for every locomotion cycle (i.e., from an IC to the next IC) normalized for 100% of the SCI-control group before (gray) and after (black) surgical intervention. Solid lines represent the mean and dashed lines the standard deviation. Vertical lines represent TO events. Positive values correspond to flexion positions for joint plots and forward positions of the ankle relative to the hip for aperture plots. Negative values correspond to backward positions of the ankle relative to the hip for aperture plots. The aperture at the hip was defined as the angle between the hip–ankle line and the vertical.

After evaluating the movement dynamics of ankle, hip, and knee joints, height of the iliac crest, and aperture at the hip (Appendix A Figure A1), we considered flexion at the knee joint as the most valid kinematic measure of controlled limb movement (Figure 4b,d). In contrast, a high value of angular movement at the ankle, for instance, did not indicate good recovery because a lack of motor control could either result in only small movements but also cause complete flexion at this joint (near 180°). Movement of the knee joint was reduced after SCI and improved with some bmSCs injections, but statistical evaluation of the treated SCI groups fell just short of the required level of significance [ANOVA, F (4, 57) = 2.22, *p* = 0.07].

The parameter of forelimb/hindlimb coordination was qualitatively represented in the open field analysis (BBB scales 11–14: none—occasional—frequent—consistent). This was quantified with kinematic analysis by calculating the percentage of forelimb cycles that coincided with one step of the ipsilateral hind limb (Figure 4e). This parameter fell from 100% in non-injured animal to 62% at 5 W after SCI (control group) and reached 75% in rats treated with the high dose of fresh bmSCs. Given the group variability, however, differences between treatment groups were not quite significant [ANOVA, sham group not included, F (4, 57) = 2.45, *p* = 0.05]. When evaluating the individual rats, our data showed a strong correlation between the BBB scores and forelimb/hindlimb coordination, evaluated from videos (Pearson r = 0.81, *p* < 0.0001), thus corroborating the validity of the different data seta.

Finally, synchronized video analysis with two cameras allowed us to evaluate coordination between the hind limbs. During normal walking, one hind paw supports the weight of the animal most of the time while the other one is elevated (monopodal support). In the remaining time, both hind paws have ground contact (rats never jumped in the setup). Since lesioned rats drag their feet and are less able to support their body weight, they display less monopodal support. This parameter decreased from 70% in non-injured rats to 29% after lesion (SCI-control) and was significantly affected by treatment [ANOVA, sham group not included, F (4, 57) = 2.99, *p* < 0.05]. Higher doses of bmSCs recovered monopodal support to 49% (SCI-bmSC-25) and 50% (SCI-bmSC-25r), which was significant (*p* < 0.5) for this group. Again, when evaluating individual rats, there was a strong correlation between the BBB scores and monopodal support (r = 0.76, *p* < 0.0001).

### 2.3. Histological Evaluation of Tissue Preservation

One hypothetical explanation for the positive effect of injected bmSCs on functional recovery is that they reduce cellular degeneration. At 6 W after SCI, when histological investigations were performed, this was indeed observed.

#### 2.3.1. Lesion Area

**Figure 5:**
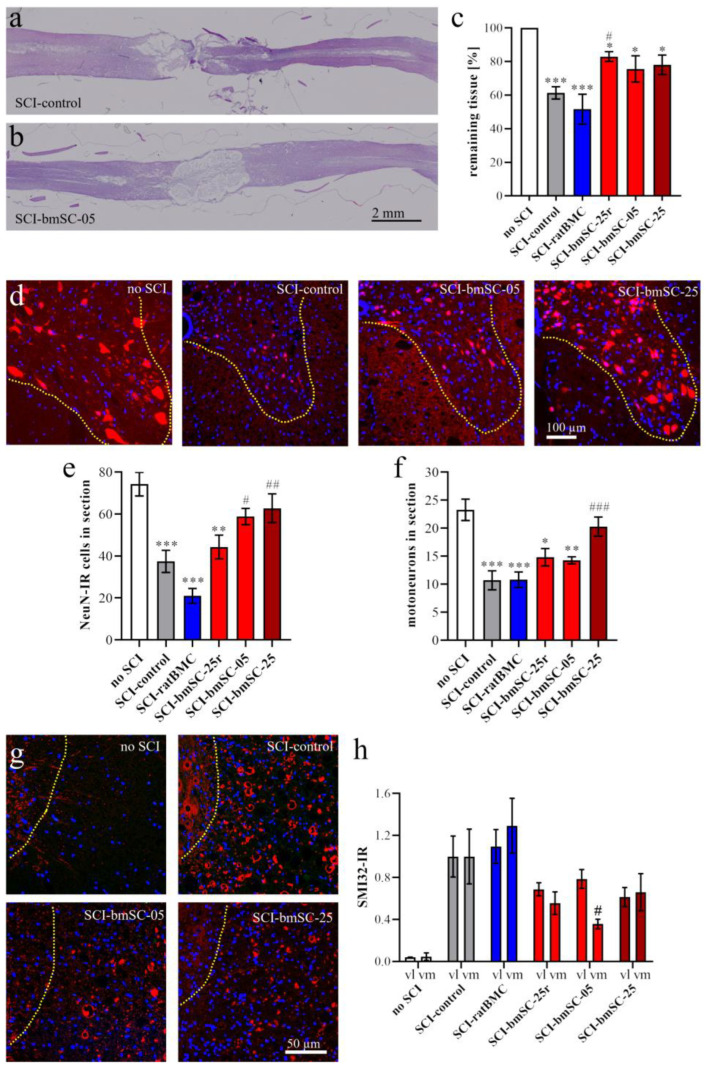
**Tissue preservation after SCI.** After 6 W, spinal cord tissue was embedded in paraffin for evaluation with histology and immunofluorescence. (**a**,**b**) Horizontal spinal cord sections at a depth of one third from the dorsal surface containing the lesion area. Representative sections of a rat without treatment ((**a**), SCI-control) and a rat treated with 500,000 fresh bmSCs ((**b**), SCI-bmSC-05), which were stained with H&E are shown. (**c**) The amount of preserved tissue was expressed as percentage of the theoretically expected non-lesioned area (no SCI). (**d**) In transverse sections of the ventral spinal cords at 4 mm caudal of the lesion center, neurons were stained with NeuN antibody (red) combined with Hoechst-33342 (blue). The border of grey matter is indicated by the dotted line. (**e**) The number of all NeuN-positive cells in the ventral horn areas, counted automatically, revealed strong neurodegeneration after SCI and significant differences in rats treated with fresh bmSCs. (**f**) Manual cell counts of motoneurons in the ventral horn areas also showed a reduction after SCI, which was significantly diminished in the SCI-bmSC-25 group. (**g**) Axonal demyelination/degeneration was visualized with SMI32 staining (red) and evaluated in the ventrolateral (shown) and ventromedial white matter at 4 mm caudal of the lesion site; nuclear staining as in (**d**). (**h**) Quantification of SMI32-IR in the different treatment groups. The left columns of each treatment condition represent data in ventrolateral (vl) and the right columns in ventromedial (vm) white matter. Data were normalized to the SCI-control group. The experimental groups are the same as explained for Figure 2, *, #: *p* < 0.05, **, ##: *p* < 0.01, and ***, ###: *p* < 0.001.

Using H&E staining of horizontal paraffin sections through the dorsal third of the spinal cords (Figure 5a,b), we measured the extent of preserved tissue in the 1 cm segment around the lesion site (Figure 5c), as well as the anterior–posterior extension of the pathological alteration of the tissue. Following SCI, there was a strong degradation leading to about 40% loss of the dorsal spinal cord tissue around the lesion center. Treatment had a significant benefit on tissue loss [ANOVA, F (4, 26) = 4.88, *p* < 0.01], which was less severe in the groups treated with human bmSCs. The difference from the non-treated controls was significant for the SCI-bmSC-25r group (Dunnett’s test, *p* < 0.05). When the means of each group were compared with the expected non-lesion area, the tissue loss was still significant in all SCI groups (one sample *t*-tests). The extension of the cyst was lowest in the SCI-bmSC-25 group (4.4 +/− 2.5 mm; mean +/− SD) compared to the SCI-control group (5.9 +/− 1.4 mm), but differences between groups were not significant [ANOVA, F (4, 29) = 1.29, *p* > 0.1]. On the basis of individual rats, we found a positive correlation between tissue preservation and behavioral recovery as assessed in the open field (Pearson r = 0.37, *p* < 0.05), indicating that better tissue preservation may partly explain motor performance.

#### 2.3.2. Neurons

Following horizontal microtome sectioning, tissue was re-embedded in paraffin, and 3 µm transverse sections of the remaining ventral 2/3 of spinal cord were made at 4 mm posterior to the lesion center in order to appreciate effects on the survival of motoneurons distal to the lesion site (Figure 5d). Using an automatic algorithm, NeuN positive cells were counted in the ventral horn areas (Figure 5e). Their numbers were reduced by half after SCI, and treatment had a significant effect [ANOVA, F (5, 49) = 13.3, *p* < 0.001], with rescue of neurons occurring in the rats treated with the lower and higher dose of fresh bmSCs (post hoc Dunnett’s test). Based on their morphology, motoneurons were counted manually (Figure 5f). Their numbers, too, were significantly affected by cellular treatment [ANOVA, F (5, 50) = 8.74, *p* < 0.001], and in this case we found a significant improvement in cell survival in the SCI-bmSC-25 group.

#### 2.3.3. Fiber Degeneration

Axonal demyelination is associated with dephosphorylation of neurofilament proteins in white matter [13]. This was visualized with a SMI32 antibody, which produced very low IR in the white matter of non-injured animals. After SCI, the SMI32-IR strongly increased the posterior and anterior of the lesion site (Figure 5g). Quantitative evaluation demonstrated significant differences between treatment groups after SCI [ANOVA, treatment: F (4, 96) = 5.66, *p* < 0.001] with similar effects in ventrolateral and ventromedial white matter [location: F (1, 96) = 0.38, *p* > 0.1; interaction: F (4, 96) = 0.95, *p* > 0.1]. This indicator of axonal degeneration was lower in all SCI-bmSCs treated groups than in rats without cellular treatment.

### 2.4. Macrophage and Glial Activation

#### 2.4.1. Phagocytes

**Figure 6:**
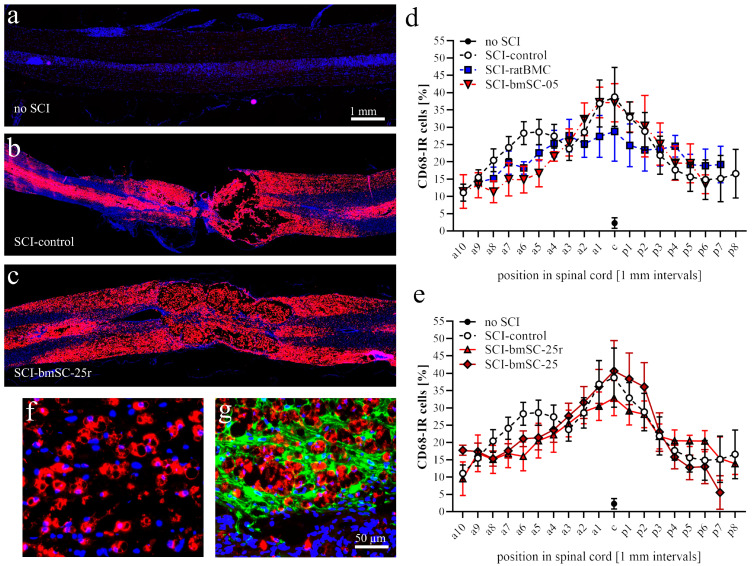
**Macrophage activity after SCI.** Macrophages were visualized with an antibody against CD68. (**a**–**c**) In horizontal sections of dorsal spinal cord, no CD68-IR macrophages were detected in sham-operated animals (**a**). Six weeks after SCI, the tissue was replete with macrophages, showing a similar distribution in all treatment groups. Representative examples of SCI-control and SCI-bmSC-25 cells are shown in (**b**,**c**). Panels a–c have the same magnification; CD68-IR (red) was combined with Hoechst-33342 nuclear staining (blue). (**d**,**e**) Quantification of CD68-IR cells as a percentage of total cell nuclei throughout the spinal cord binned at 1 mm intervals along the anterior–posterior axis in the dorsal third of the spinal cord. Groups are labeled as described in the legend to Figure 2. (**f**,**g**) Higher magnification photographs of CD68-IR cells (red) in gray matter (**f**) and at the border of the lesion center (**g**), where their location within the GFAP-IR glial scar (green) is shown. The scale bar in (**g**) indicates magnification in panels (**f**,**g**).

While treatment with bmSCs reduced tissue degeneration, the cellular and molecular mechanisms for this are largely unknown. Since a frequently suggested mode of action is the release of anti-inflammatory exosomes or paracrine factors [14], we assessed the effect of bmSC treatment on the activation of macrophages and microglia. Macrophages derived from either endogenous microglia or the circulation were labeled with a CD68 antibody (Figure 6a–c). When the number of CD68-IR cells was quantified in horizontal sections through the dorsal spinal cord, we found a strong accumulation of macrophages in the center of the lesion, a gradual decrease in the anterior and posterior directions, and almost no CD68-IR cells in the spinal cords of sham-operated rats (Figure 6d–e). Close to the lesion center, macrophages appeared within the cyst and were interspersed with scar tissue (Figure 6f,g). Two factor ANOVA demonstrated a strong effect of proximity to the lesion site in the anterior–posterior direction [F (16, 397) = 9.48, *p* < 0.0001], but among the SCI groups there was no significant effect of treatment [F (4, 397) = 1.38, n.s.] and no interaction between location and treatment [F (64, 397) = 0.47, n.s.]. With respect to individual rats, we found no significant correlation between the number of CD68-IR macrophages and sensory–motor function at 5 W (Pearson r = −0.34, n.s.).

#### 2.4.2. Microglia Morphology

**Figure 7:**
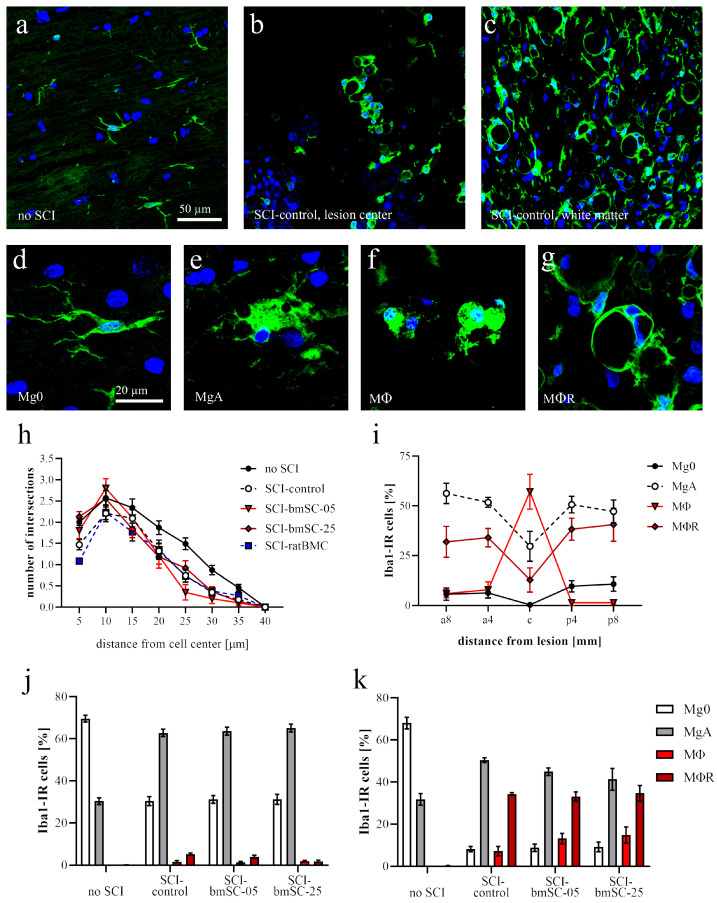
**Microglia activation after SCI.** Microglia cells were visualized with an Iba1 antibody and counterstained with Hoechst-33342. (**a**–**c**) Microscopy images of Iba1-IR cells (green) in white matter of a sham-operated rat (**a**) at 6 W after SCI in the lesion center (**b**) and in white matter at 4 mm posterior of the lesion center (**c**); same magnification in (**a**–**c**). The characteristic IF signals shown here were similar in all experimental groups with SCI. (**d**–**g**) Examples of Iba1-IR cells, which were classified based on their morphological appearance: so-called resting microglia ((**d**), Mg0), strongly activated microglia ((**e**), MgA), macrophages without any ramified processes ((**f**), MΦ), and macrophages with annular-shaped morphology ((**g**), MΦR); same magnification as in d (scale bar). (**h**) Scholl analysis of ramified microglia (Mg0 and MgA) under different treatment conditions. In sham-operated rats, microglia cells typically extended branches at 25–35 µm distance from the cell nucleus. After SCI, Iba1-IR cells had fewer and shorter ramifications, as quantified in gray matter at 4 mm posterior of the lesion center. This was similar in all treatment conditions. (**i**–**k**) Iba1-IR cells were classified according to the categories indicated above (Mg0, MgA, MΦ, MΦR). Relative frequencies of these morphologies changed with respect to distance from the lesion center ((**i**), shown for rats in the SCI-control group). At a 4 mm distance from the lesion center, most Iba1-IR cells in gray matter (**j**) resembled activated macrophages, while in white matter (**k**) the characteristic annular-shaped cells and macrophages were also frequent. Cellular treatment had no significant effect on the distribution of Iba1 morphologies.

In response to SCI, microglia cells became activated, and their morphology changed (Figure 7a–c). To evaluate whether injected bmSCs affected this response, we labeled microglia/monocytes with an Iba1 antibody and classified the IR cells into four groups (Figure 7d–g): extensively ramified microglia (Mg0), activated microglia with large cell body and few short ramifications (MgA), macrophages (MΦ), and ring-shaped macrophages (MΦR). We found this cellular morphology in white matter, where they may have formed around degenerating fiber bundles. Random samples of Iba1-positive cells with cytoplasmic processes were photographed and evaluated with Scholl analysis. In comparison with tissue from sham-operated rats, Iba-IR cells in all SCI groups had fewer ramifications, indicating microglial activation (Figure 7h). There were no differences between treatment groups. The morphological classification of Iba1-IR cells in SCI tissue showed a preponderance of MΦ in the lesion center, decreasing with distance in rostral and caudal directions. At 4 mm and 8 mm anterior and posterior, MgA were the most frequent morphological type, followed by MΦR and few MΦ and Mg0 (Figure 7i). This pattern was the same in all treatment groups. In the graphs (Figure 7j,k) we show the proportion of different Iba1-positive cells in tissue from sham-operated rats, SCI-control, SCI-bmSC-05, and SCI-bmSC-25 treatment groups at 4–8 mm posterior of the lesion center in gray (Figure 7j) and white matter (Figure 7k). Spinal cord injury caused a significant change in the morphological distribution of Iba1-IR cells, but there was no significant effect of treatment.

#### 2.4.3. Astrogliosis

**Figure 8:**
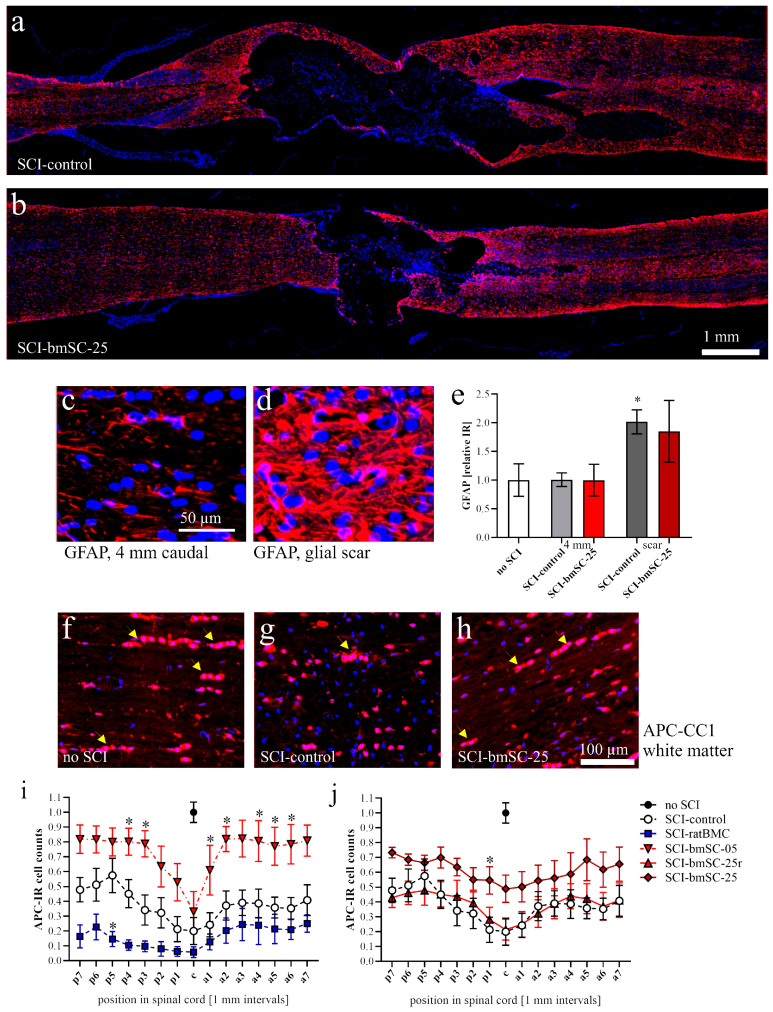
**Astrocytes and oligodendrocytes at 6 W after SCI.** (**a**,**b**) Microscopy mosaic images (20× objective, CellSens/QuPath) of horizontal spinal cord sections stained with GFAP antibody (red) and Hoechst-33342 (blue). (**c**,**d**) Examples of GFAP-positive astrocytes 4 mm posterior to the lesion center (**c**) and of scar tissue in the SCI-control group (**d**). (**e**) Quantification of GFAP IR in white matter of sham-operated rats after SCI at 4 mm posterior to the lesion site, and in the scar area around the lesion site in groups SCI-control and SCI-bmSC-25, * *p* < 0.05. (**f**–**h**) Examples of APC-positive oligodendrocytes in white matter at 4 mm distance from the lesion center, indicated with arrow heads. (**i**,**j**) Quantification of the number of APC-IR nuclei in white matter at 1 mm intervals anterior and posterior to the lesion center. Data were normalized to cell counts in sham injured rats. In panels (**i**,**j**), significant differences (*p* < 0.05 or *p* < 0.01) from the SCI-control group are indicated with single asterisks. Experimental groups are labeled as in Figure 2.

The intensity of GFAP staining was evaluated in horizontal sections located in the dorsal third of the spinal cord (Figure 8a,b). In the white matter at a 4 mm distance from the lesion site at 6 W after SCI, GFAP-IR was not different from that in animals with laminectomy only (Figure 8c,e; *t*-tests, *p* > 0.1). To assess the effect on the astrocytic scar, ROI of 1 mm^2^ size were drawn to cover the GFAP-positive area around the lesion center, which itself was devoid of astrocytes. Here, the GFAP intensity in rats with SCI was two-fold higher than in sham-operated animals (*p* < 0.05). We found no treatment effect (Figure 8d,e).

#### 2.4.4. Oligodendrocytes

In consecutive sections, oligodendrocytes were visualized using an antibody, which marks the nuclei of these glial cells (APC-clone CC1, Figure 8f–h). This staining pattern allowed us to identify and count APC-IR cell bodies in 1 mm intervals in the white matter from rostral to caudal of the lesion center. Location as well as treatment were significant for the number of oligodendrocytes at 6 W after SCI [ANOVA (sham group excluded), location: F (14, 341) = 3.42, *p* < 0.0001; treatment: F (4, 341): 67.7, *p* < 0.0001; interaction: F (56, 341) = 0.35, *p* > 0.1]. As expected, the density of oligodendrocytes was lowest near the lesion center. It increased with distance, although within the investigated area cell numbers did not reach the level found in animals without SCI (Figure 8i,j). In rats treated with fresh human bmSCs, the number of oligodendrocytes was significantly higher than in the SCI-control group.

### 2.5. Presence of Injected Human bmSCs in the Rat Spinal Cord

**Figure A2:**
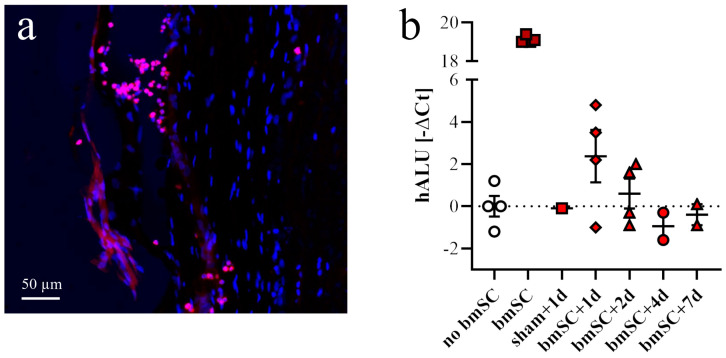
**Presence of injected bmSCs in the rat spinal cord.** (**a**) At 1 day after intrathecal injection, human bmSCs were detected with an antibody against human mitochondria. The IR cells were located near the meninges close to the lesion site. No such signals were found in spinal cord tissue prepared later after injection or in animals that had not received bmSC treatment. (**b**) Amplification of human DNA in spinal cord extracts from rats with SCI; shown are differences in threshold cycles between experimental groups and negative controls (n = 2 rats/group, 2 PCR experiments/rat, all data shown): rats with SCI but no cell injection (no bmSC); extracts from human bmSCs, used as a positive control (bmSC); rats with laminectomy, bmSC injection, preparation at 1 dpi (sham + 1d); all other groups are rats with SCI, preparation at 2 dpi (bmSC + 2d), preparation at 4 dpi (bmSC + 4d) and preparation at 7 dpi (bmSC + 7d).

Twenty-six animals were operated with the purpose to detect the presence of injected bmSCs within the spinal cord after SCI (Figure 1b). At 1, 2, and 4 dpi each, three rats were sacrificed and spinal cord sections were stained with a fluorescence-labeled antibody against human mitochondria. Only in tissue prepared at 1 dpo did we find IR-cells. These were located near the meninges of the spinal cord near the lesion site (Appendix B Figure A2a). In animals sacrificed at 2 dpi and 4 dpi, at other positions, and in animals with SCI that did not receive a bmSCs injection, no human bmSCs were detected.

As an alternative approach to verify the presence of injected human cells, DNA was extracted from the spinal cord tissue of treated rats. Using primers against human ALU elements, we were able to amplify human DNA in samples prepared at 1 dpi but not at 2 dpi, 4 dpi, 7 dpi, or in tissue from rats without bmSCs injection (Appendix B Figure A2b). Although the absence of evidence is never conclusive, our preliminary investigation indicate that injected human cells disappeared rapidly.

## 3. Discussion

The present study found a significant benefit in function after treatment with human bmSCs in a rat model of SCI. Cells were prepared from the bone marrow of healthy donors using a negative selection procedure without cultivation (NeuroCells^TM^). Following intrathecal injection of 0.5 × 10^6^ or 2.5 × 10^6^ cells one week after lesion, the animals were observed for five more weeks. Treatment with the high dose but not with the low dose resulted in a significantly better motor recovery in comparison with saline or cultivated stromal cells from rat bone marrow.

### 3.1. Timing of Treatment after SCI

One objective of the present study was to determine whether cellular treatment in the post-acute or early chronic phase is more effective than application immediately after SCI. We reasoned that cell survival would be better when the immediate inflammatory peak had subsided. In rodents, the highest increase in the pro-inflammatory cytokines IL-1β, TNFα, IL-6, INFγ, and chemokine CCL-2 (MCP-1) occurs during the first week after SCI, while the anti-inflammatory cytokine IL-10 peaks later, at 7 dpo [15,16]. By injecting bmSCs at 7 dpo, we also expected to have a higher probability of influencing the activity of peripheral macrophages, lymphocytes, and pericyte-derived cells [15,17]. From a clinical point of view [4,18], a delayed application of bmSCs is more realistic than intervention at the time or shortly after lesion, as it is often practiced in animal models [2,19]. Two previous studies included rats which received cisterna magna injections of human bmSCs at 1–2 h after lesion [11,12]). Since these experiments otherwise followed the same protocols and were performed by the same investigators, the results may be compared. Contrary to our expectation, the functional benefit of treatment increased very little with the delayed application: At 6 W after SCI, the improvement in sensory–motor function was 1.2 BBB-scores when 2.3 × 10^6^ cells were given immediately after SCI and 1.6 BBB-scores when 2.5 × 10^6^ cells were injected one week after SCI. In these cases, the bmSCs had been stored in liquid nitrogen and reconstituted before application. However, the same number of viable cells when applied without cryopreservation resulted in an improvement of 3.4 BBB-scores. Therefore, the use of freshly prepared cells appears to be more important for the outcome than the time of application. Osaka and coworkers systematically tested different time points of cell treatment in a rat model of contusion SCI. They used intravenous injections of autologous mesenchymal stem cells [20]. Interestingly, this study showed a rather broad therapeutic window with similar results of motor recovery when cells were injected at 1, 3, or 7 dpo. Effects of treatment decreased progressively with delayed administration, but even cell injection at 28 dpo produced a functional benefit two weeks later. More recent data with i.v. injection of mesenchymal stem cells at 10 W post SCI indicate that cellular treatment can be beneficial even when a cystic cavity has already formed [21,22].

### 3.2. Non-Manipulated bmSCs vs. bmSCs

An expansion of bone marrow-derived cells in culture allows for the production of much higher cell numbers and also provides more flexibility with respect to clinical administration. Both are serious issues because the number of bmSCs extracted from SCI patients can be insufficient and the logistical coordination between surgical extraction, purification, and injection of the cells is challenging (our own experience: EudraCT 2018-000805-22). Therefore, the justification for using non-manipulated, fresh bmSCs rests primarily on their higher biological activity. It is difficult to test this without comparing fresh bmSCs (NeuroCells^TM^) with cultivated bmSCs in the same study. Nonetheless, one excellent review [19] provides detailed data from preclinical experiments with cell therapy of SCI. In nine studies documenting transplantation of cultivated human bmSCs in rat SCI models, only two reached improvements of more than 1.5 BBB-scores. Some publications during the last decade found stronger effects with human endometrial stem cells: ΔBBB ≈ 3 [23] and neuron-like induced fibroblasts: ΔBBB = 3.1 [24]; all data points at 5 W. In the present study, the group with highest dose of fresh bmSCs reached a difference of 3.4 BBB-scores compared to saline treated controls. For bone marrow-derived stromal cells, our data support the hypothesis that non-manipulated, fresh bmSCs are therapeutically better than cells expanded in vitro; however, other sources of human stem cells may provide better alternatives for the clinical use, even with expansion in culture. The present data also indicate that the dose is relevant. In most functional tests, we found 2.5 × 10^6^ cells to produce better functional recovery than 1/5 of that number, and most differences with the control groups were only significant with the higher dose. Only the lower concentration of bmSCs can directly be compared with the ratBMC preparation; however, the complete absence of a treatment effect in this case makes it unlikely that a higher dose of these cells would have been superior to the high dose bmSC treatment. Our human bmSCs preparation has a low immunogenic activity [8,11], which may account for the absence of a systemic rejection. The absence of a local immune response to the cells in the chronic phase may also be due to their rapid elimination or because only small numbers had migrated to the lesion site.

### 3.3. Behavioral Analysis

Since the primary outcome measure in this study was recovery of sensory–motor function, we supplemented the open field evaluation, which is the standard procedure in rat models of SCI [22,25], with sensory tests and kinematics analysis. For investigations of severe SCI we consider the open field evaluation of motor function with the BBB scale [26] to be the most comprehensive measure because it includes mobility at all hind limb joints, paw positioning on the ground, toe clearance, coordination between hind- and forelimbs, gait stability, and covers all phases of recovery from complete paralysis to normal gait. Since the empirical scale is incremental, each individual score provides information on all of the listed parameters. Despite the fact that the BBB rating is only an ordinal scale, it is standard practice to calculate means and use parametric statistics. The only drawback of the method is that its validity depends heavily on the experience of the evaluating scientists. The CatWalk provides a more objective measure of some parameters that are assessed in the open field, but the animals have to be able to walk [27,28]. As a quantitative method that can be used when rats are not capable of plantar stepping, we chose kinematic analysis using two synchronized cameras and open source software (Kinovea, version 0.9.5). Our data from video analysis at 5 W correlate well with the BBB scores, which would indicate that the kinematics analysis was not truly necessary for the conclusion of this study. On the other hand, the correlation between some of the kinematic parameters was lower, suggesting that not all neurological correlates of behavior recovered in parallel. For instance, the correlation between forelimb/hindlimb coordination and monopodal support was only r = 0.66. Treatment with high doses of bmSCs improved coordination between the hind limbs (monopodal support) but had only a small, non-significant effect on coordination between forelimbs and hindlimbs.

Tests with mechanical (von Frey) and heat stimulation (Hargreaves) are primarily designed to measure pain thresholds [29]. When the animals are not capable of moving their paws, which is the case during the first weeks after a severe contusion SCI, these tests cannot be applied. In the chronic phase, however, this is possible, and by comparing the results with the response range of non-injured animals, we consider that these tests also valid to detect a sensory deficit, as indicated by increased paw withdrawal thresholds. This was observed in rats of the SCI-control group but not in those treated with the high dose of fresh bmSCs.

### 3.4. Mechanism of Action

The present investigation did not focus on the mechanism of action of bmSCs, and we limit ourselves to a few comments. Since cellular treatment occurred after the blood–brain barrier had been breached, the invasion of hematogenous neutrophils and macrophages [15] was unlikely to be influenced by the treatment. At this post-acute stage, the inflammatory reaction, though already decreasing, is still a prevailing process and typically continues into a chronic stage. This implies not only cytotoxic effects but may also compromise the phagocytosis ability of macrophages [30]. We hypothesize that the injected bmSCs are beneficial for reducing these pathological processes, because anti-inflammatory effects of these cells were observed in previous experiments, which included analysis in the next days after treatment [12,14]. In the chronic phase, remyelination of demyelinated axons, axonal regeneration, axonal sprouting, angiogenesis, and enhancement of neuronal plasticity have been suggested to be positively affected by bmSC treatment [22]. It appears, however, that effects do not last. Similar to many other publications, we observed a treatment effect only up to a few weeks after application. In published SCI studies with severe contusion injury, animals never even remotely show a complete recovery of motor functions [2,19,25]. In the present experiments, we detected the human bmSCs in the rat spinal cord tissue one day after their injection (homing effect) but not at later stages. From the mere absence of evidence, we may not exclude that some of the treatment effects are due to continuing activity of surviving bmSCs, but it is not likely. Thus, therapeutic benefits of bmSCs application after SCI may be enhanced with a combination strategy that supports survival and integration of the injected cells. In the case of bmSC treatment in the chronic phase after SCI, a therapeutic benefit seems to be due to remyelination of surviving axons [21,22]. If this is the dominant effect, the treatment would depend on the sparing of axons within the spinal cord, and we would expect less or no functional benefit in cases of complete spinal cord transection. Fortunately, most clinical cases do not fall in this category.

## 4. Materials and Methods

### 4.1. Experimental Animals

The experimental protocol, surgical procedures, and postoperative care were reviewed by the ethics committee for Animal Care of the Hospital Nacional de Parapléjicos (163CEEA/2017) and approved by the regulatory authority of Castilla-la Mancha (ref. 210498, following EU directive 2010/63/EU). The study was conducted with male Wistar rats (*Rattus norwegicus*) from the breeding facility of the hospital, which had a body weight of 240 to 290 g at the day of surgery. Since the gain in body weight, which may affect motor performance, is different in male and female rats, mixed sex groups would have required a larger number of animals. Male rats were chosen to avoid the potential influence of changing hormone levels during the estrous cycle either at the time of SCI or at the time of treatment one week later.

Standard housing conditions consisted of a 12 h light/dark cycle, humidity 40–60%, and temperature 22 °C with *ad libitum* access to food and water. A total of 68 animals with SCI entered the study, 42 in the principal investigation and 26 in an experiment to trace injected stem cells. In addition, the spinal cords of 3 rats with laminectomy only (sham) and 2 rats without surgery were processed for comparison of histological results or PCR (Figure 1a). Sample size calculation was based on previous experience and the availability of fresh human stem cells [12] (α = 0.05, β = 0.2, BBB score as primary outcome with expected d = 3, SD = 2, attrition 10%). Since rats had the same sex, and similar age and body weight, no randomization was necessary to allocate them to the different groups.

### 4.2. Surgical Procedures and Postoperative Treatment

For spinal cord injury, anesthesia consisted of 2.5% isoflurane/97.5% oxygen at 0.5 L/min for SCI with one s.c. injection of buprenorphine 0.05 mg/kg 15 min before surgery. Corneal dehydration was prevented with ophthalmic ointment (Lubrithal). Following laminectomy at thoracic level T9, a spinal cord contusion of 2 N (200 Kdyn, zero dwell time) was performed with the Infinite Horizon spinal cord impactor. The procedure was checked visually (hematoma) and by monitoring the displacement/time and force/time plots. After the operation, animals received 2 × 1.5 mL isotonic saline s.c. and antibiotic 5 mg/kg marbofloxacin (10 mg/mL, s.c.). Postoperative care, including analgesics, antibiotic treatment, and manual voiding of the bladder, was performed as described previously [11,12].

One week after SCI, the animals received one injection into the cisterna magna. This was done with the animals under anesthesia with one i. p. injection of ketamine 50 mg/kg combined with xylazine 5 mg/kg. For the transplantation, the anesthetized animals were positioned in a stereotactic frame, and the atlanto-occipital membrane was exposed and penetrated with a pointed scalpel blade. A Fogarty arterial embolectomy catheter (d = 0.67 mm) was then inserted 3–5 mm into the cisterna magna and the cell suspension was slowly infused with a syringe pump (150 µL/5 min). While the rat was being prepared by one researcher, a second person suspended 0.5 or 2.5 million cells for injection based on cytometric counting of cell numbers and viability. Euthanasia at the end of the study was induced by i. p. injection of 100 mg/kg sodium pentobarbital.

### 4.3. Experimental Groups

Animals were assigned to five experimental groups, which received the same SCI but differed in the treatment procedure (Figure 1a). The Group *SCI-control* received one injection of 150 µL saline into the cisterna magna at 7 days after SCI (7 dpo). Animals of all other treatment groups were given 150 µL cell suspension injected into the cisterna magna at 7 dpo. Group *SCI-bmSC-05* received 0.5 × 10^6^ fresh bmSCs, Group *SCI-bmSC-25* received 2.5 × 10^6^ fresh bmSCs, and Group *SCI-bmSC-25r* received 2.5 × 10^6^ bmSCs which were stored in liquid nitrogen and resuspended immediately before application. A second control, Group *SCI-ratBMC*, was injected with 0.5 × 10^6^ stromal cells from rat bone marrow. Cell numbers refer to live cells as determined with cytometry prior to injection. Spinal cord sections from three rats that had T9 laminectomy but underwent no contusion injury were used for comparing histological data.

For the purpose of detecting injected cells, two additional groups of rats with SCI received one injection of 2.5 × 10^6^ bmSCs (stored in liquid nitrogen and resuspended) or the same volume of saline into the cisterna magna. Animals were sacrificed 1, 2, 4, or 7 days after bmSCs injection (dpi) into the cisterna magna (Figure 1b). Histological staining to detect implanted cells was also carried out with tissue from SCI-control and the bmSC-treated groups sacrificed after 6 weeks. The spinal cords of two rats without surgery were prepared to control for false positive PCR results.

### 4.4. Preparation of bmSCs

Bone marrow-derived stromal cells from human donors. Human bmSCs for SCI treatment were prepared as described previously [11,26]. Following bone marrow extraction from the donor, the procedure consisted of Ficoll density gradient centrifugation followed by the elimination of B-cells (CD20), T-cells (CD3), monocytes (CD14), and natural killer cells (CD56) using antibody-based cell sorting with magnetic beads under GMP conditions. Cells were not expanded by cultivation (Neuroplast BV, patent WO2015/059300A1). All procedures for collection of human bone marrow were approved by the ethics committee of Maastricht University Medical Center (METC 13-2-032). The viability and cell type composition of each batch were analyzed with flow cytometry (CD34, CD271, CD90, CD105, CD73). For the present study, bmSCs were prepared at the Neuroplast facility in Geleen, NL, and shipped in a cooled, temperature-controlled container within 24 h to the HNP Toledo, using the same procedure as followed for a clinical trial (EudraCT 2018-000805-22). One experimental group of animals received bmSCs that were cryoprotected with DMSO, frozen in liquid nitrogen, shipped on dry ice to Toledo, Spain, and then stored in liquid nitrogen until use. Cell viability (exclusion of 7-amino-actinomycin D, cytometry) was determined immediately before application in vivo.

Stromal cells from rat bone marrow. To control for effects of intrathecal cell injection, we prepared cells from the bone marrow of adult rats (ratBMC). Cells were obtained from femur and tibia bones that were aseptically harvested from male rats euthanized with an overdose of sodium pentobarbital. Bone marrow was obtained by rinsing each harvested bone with growth medium (Dulbecco’s Modified Eagle medium, DMEM; GIBCO) using a 3 mL syringe. They were separated by centrifugation at 2000 rpm at RT for 45 min on a 3 mL Ficoll gradient. The obtained cells were seeded in a 75 cm^2^ culture flask with 10 mL of DMEM, 20% heat-inactivated fetal bovine serum, 2 mM L-glutamine, streptomycin/penicillin (Sigma P4458, 1/100), and nonessential amino acids (Lonza 3-114E, 1/100, other cell culture reagents from GIBCO). Cells were maintained in a water-jacketed incubator at 37 °C with 5% CO_2_ until a monolayer of feeding cells was formed, approximately three weeks. Afterwards, a new batch was prepared using the same procedure and cultured on top of feeding cells for four passages. These ratBMC were cryopreserved with DMSO and stored in liquid nitrogen until use.

### 4.5. Evaluation of Locomotor Functions

Open field assessment. Recovery of limb movements was evaluated in the open field using the Basso–Beattie–Bresnahan (BBB) locomotor function scale [26]. This was performed before SCI surgery (baseline), then at 2 dpo, 4 dpo, 7 dpo (i.e., before treatment), at 9 dpo, 11 dpo, 14 dpo, and subsequently once per week until six weeks after SCI. At the beginning, we established a criterion of BBB ≤ 1 at 2 dpo for inclusion in the study because a higher score was considered to indicate incomplete SCI. At 7 dpo, before treatment, a negative criterion (BBB ≤ 1) was applied to exclude rats with excessive lesions. Scoring was performed independently by two investigators who were blinded with respect to the treatment of the individual animals. The average score was given if, after discussing their evaluation, both investigators assigned different ratings.

RotaRod test. Complementary to the open field assessment, the rats were subjected to the RotaRod test (Ugo Basile SRL, Gemonio, Italy). In this task, rats are positioned on a slowly rotating rod, which obliges them to use their hind legs to maintain their balance. During tests, the rotation speed was accelerated from 5 rpm to 15 rpm over a period of 5 min, and the time was recorded that the rats were able to stay on the rotating rod (two repetitions, separated by a break of ≥15 min). In six training sessions of 5 min each, at three consecutive days before SCI, all rats learned this task at a constant speed of 5 rpm of the rotating rod. After confirming at 4 dpo that none of the rats were capable of weight-supported stepping, the test was administered for the first time at 7 dpo and then once per week until the end of the study.

Video recordings. At 5 W, when all rats showed hind limb movements due to spontaneous or treatment-induced recovery, their motor performance was quantified with video recordings. Rats were trained to move along a 1.3 m long pathway with walls of transparent plexiglass at the sides and equipped with a dark chamber at the end. While running, the rats were being filmed by two synchronized digital cameras (MotionScope. Redlake MASD Inc., San Diego, CA, USA) at 125 fps, resolution 480 × 420 pixels, black and white. Capturing of the images was triggered externally. To later evaluate angular movements at the ankle, knee, and hip joints, black marks were painted on the shaved skin at these positions, at the end of the fifth digit, at the cervical crest, and at the ankle of the forepaws. With each rat, three successful runs were evaluated.

Kinematic analysis. For image processing we used Kinovea 0.9.5 freeware (https://www.kinovea.org (accessed on 17 January 2022)). The times of initial contact (IC) and detachment (toe off, TO) of the paws were marked manually. Cartesian coordinates were calculated using automatic detection of the skin markers. The following data were acquired from every video frame during the rats’ movements: positions and angles of the hip, knee, and ankle of the hind limbs, as well as step length and speed (Appendix A Figure A1). For the analysis of motor recovery after SCI, we selected the following four quantitative parameters. (1) Stepping frequency: Number of cycles (IC-TO-IC) of the hindlimbs. (2) Hindlimb mobility: Mean angle between maximal and minimal flexion of the knee joint. (3) Forelimb/hindlimb-coordination: For each cycle of the forelimb, it was determined whether there was a corresponding cycle of the ipsilateral hindlimb (IC; yes/no). (4) Monopodal support: Percent of time when only one of the hindlimbs touched the ground.

### 4.6. Tests of Allodynia/Hyperalgesia and Sensory Loss

Von Frey test. The response to mechanical stimulation was evaluated using a dynamic plantar aesthesiometer (von Frey test 37550; Ugo Basile, Gemonio, Italy). For each hind leg, a paw withdrawal threshold (PWT) was determined up to a maximum force of 50 g. This was performed five times with at least five-minute intervals between tests. The lowest and highest values of these readings were excluded and then the mean was calculated as the PWT.

Hargreaves method. In the same way, we determined the response to heat stimulation using an infrared beam (Hargreaves test 37570; Ugo Basile). Here, the PWT was measured as the latency [sec] until the stimulated paw was moved. The intensity of stimulation was set at 40.

Both tests were administered five weeks after SCI, when all animals were physically able to respond to the stimulation. To evaluate the responses, we established the mean PWT of five rats without SCI. A response within 2 SD above and below this threshold was considered normal. When the PWT of a limb was more than 2 SD below the mean of non-injured rats, it was considered a sign of neuropathic pain. A PWT of more than 2 SD above normal indicated a sensory deficit. All animals responded to stimulation.

### 4.7. Tissue Preparation and Histological Staining

Rats were sacrificed with an overdose of sodium pentobarbital followed by transcardial perfusion with PBS and 4% paraformaldehyde/PB (PFA). We dissected spinal cord segments of 2 cm length centered at the lesion site. The tissue was post-fixed for 24 h in PFA at 4 °C, stored at 4 °C in PFA for 1–3 days, then dehydrated, and embedded in paraffin (Leica EG1150H; Leica, Wetzlar, Germany). Horizontal sections of 3 μm were cut from dorsal to ventral using a Leica RM2265 microtome. They were mounted on polylysine-coated glass slides (Superfrost Plus) and stored at room temperature (RT).

To evaluate the extent of the lesion site, one dorsal section at the horizontal level above the central gray matter was selected and stained with hematoxylin/eosin (H&E) following a standard protocol. Stained sections were again dehydrated and coverslipped with Histomount. Immunofluorescence staining (IF) was performed on the adjacent sections, all located in the dorsal spinal cord. For the assessment of neuron survival and axonal degeneration posterior to the lesion site, the remaining tissue was re-embedded in paraffin to prepare 3 μm transverse sections at 4 mm posterior to the lesion site.

### 4.8. Immunofluorescence Staining

For IF staining, sections were rehydrated and incubated for 30 min at 90 °C (water bath) in 10 mM Na citrate, pH 6.0, for antigen retrieval. Background staining was reduced by blocking 30 min at room temperature (RT) with 5% normal goat serum/0.1% Tween 20 in Tris-buffered saline (TBS-T). Sections were incubated with primary antibodies for 12 h at 4 °C in a humidified chamber and for 1 h at RT with fluorescence-labeled secondary antibodies using a hydrophobic marker pen and 200 μL/slide. Nuclei were stained with 10 μg/mL Hoechst-33342 for 15 min at RT. Sections were cover slipped with ImmuMount (Thermo Scientific, Alcobendas, Spain). The following primary antibodies were used in double staining experiments: polyclonal rabbit anti-GFAP (Sigma G9269; 1/500), polyclonal guinea pig anti-Iba1 (Synaptic systems 234004; 1/500), monoclonal mouse antibodies against CD68 (Serotec MCA341R1; 1/250), GFAP (BD Pharmingen 556327; 1/500), NeuN (Chemicon MAB377; 1/200), APC clone CC1 (Sigma MABC200, 1/300), and SMI32 (Covance 32R100, 1/2000). Antibodies against these epitopes were intended to visualize astrocytes and glial scar (GFAP), microglia and macrophages (Iba1), active phagocytes (CD68), neurons (NeuN), oligodendrocytes (APC clone CC1), and non-phosphorylated neurofilament in degenerating axons (SMI32).

To detect injected human bmSCs, sections from dorsal and ventral spinal cord were stained with a fluorescence-labeled mouse monoclonal antibody against human mitochondria (Merck MAB1273C3; 1/200). Secondary antibodies (for all other primary antibodies) were labeled with fluorescent dyes: goat anti-guinea pig IgG, Alexa-488 (Invitrogen A11073; 1/400), goat anti-rabbit IgG, Alexa-488 (Invitrogen A11008; 1/1000), goat anti-mouse IgG, Alexa-594 (Invitrogen A11005; 1/1000), goat anti-mouse IgG, and Alexa-488 (Jackson 115-545003; 1/1000).

### 4.9. Microscopy and Image Analysis

Histological and immunofluorescence staining experiments were evaluated using (1) a Leica DM5000B epifluorescence microscope (20×, 40× objectives), (2) an Olympus automated IX83 microscope (UPLXAPO 10×/0.40, 20×/0.80, 40×/0.95 objectives) equipped with *ScanR* and *CellSens Dimensions* software (version 0.3.2) for automatic capture and mosaic composition of multiple images, or (3) an Olympus BX61 stereotaxic microscope (2× objective), depending on the task. Exposure conditions were always kept constant for quantitative evaluation of fluorescence images. Photographs were analyzed using *QuPath Bioimage Analysis* (https://qupath.github.io/ (accessed on 10 May 2023)) and *Fiji Image-J* (version 2.15.0), applying the same brightness/contrast adjustments and threshold values for each marker.

Tissue preservation. Horizontal paraffin sections through the dorsal third of the spinal cord were stained with hematoxylin and eosin (H&E, standard procedure) and photographed with the stereotaxic microscope. At 6 W, two measures were taken at (a) the anterior–posterior extension of the lesion site and (b) the remaining tissue area as a percentage of expected tissue without lesion. For this, a 1 cm segment centered on the lesion site was chosen as the ROI. The expected area of tissue without lesion was calculated using the breadths of the section at the anterior and posterior end of the ROI. The area of remaining tissue was measured using *Image-J*.

Macrophages. Using QuPath, automatic cell detection based on nuclear staining and CD68-IF was performed in dorsal spinal cord sections. We determined the percentage of CD68-positive cells in 1 mm bins from 9 mm anterior to 9 mm posterior of the lesion site. In addition, the intensity of CD68-IF was measured in the same areas (*Image-J*, integrated density) following background subtraction.

Microglia. Based on their morphology, Iba1-IR cells were classified as nonactivated microglia (with cellular processes, Mg0), activated microglia (large soma with few processes, MgA), phagocytes (smaller round soma without cytoplasmic extension, MΦ), or annular shaped macrophages (ring like structures surrounding empty spaces, MΦR). At positions 8 mm anterior, 4 mm anterior of lesion center, in the lesion center, and 4 mm and 8 mm posterior of the lesion center, the number of Iba1-IR cells with these morphologies was counted in ROIs with 1 mm anterior–posterior extension. Secondly, the IF intensity was measured as for CD68. In addition, a Scholl analysis was performed on Iba1-IR cells randomly selected at 4 mm posterior of the lesion center. Cells were photographed with 40× objective, and the number of cellular processes at 5 µm radial distance from the cell nucleus was counted using the *concentric circles* plugin for *Image-J* version 2.15.0 (n = 30 cells/group).

Neurons. At 4 mm posterior of the lesion center, transverse sections of the ventral half of the spinal cord were evaluated. An ROI comprising the ventral horn gray matter was analyzed with QuPath to count the total number of NeuN-positive neurons, using automatic selection parameters of cell size and IF intensity. In addition, the number of motoneurons, based on the size of NeuN-positive cells, was counted manually by two independent observers blinded to the sample conditions. SMI32-IR (integrated density) was measured in ROIs located in the ventromedial and ventrolateral white matter of these same sections.

Astrocytes. The GFAP-IF signal was measured as integrated density (*Image-J* version 2.15.0) at positions 4 mm posterior, 4 mm anterior, and around the lesion site in a 1 mm^2^ (1.02 ± 0.03) ROI in three of the experimental groups (sham, SCI-control, SCI-bmSC-25).

Oligodendrocytes. The percentage of APC clone CC1-positive cells among all nucleated cells was assessed with QuPath using automatic selection parameters of cell size and IF intensity. The analysis was performed in horizontal sections of the dorsal spinal cord from 9 mm anterior to 9 mm posterior of the lesion site. White and gray matter were evaluated separately.

### 4.10. Detection of Human DNA in bmSCs Treated Rats

In addition to histological methods, quantitative PCR was employed to detect injected stem cells in the spinal cords of treated rats. For this, animals were sacrificed at 1, 2, 4, and 7 days after injection of bmSCs using an overdose of sodium pentobarbital. Spinal cord samples consisted of a 1 cm segment with the lesion site in the center. Of this tissue, 20 mg was homogenized in 180 µL digestion buffer (Fisher K1820-00) and the DNA was extracted according to the manufacturer’s instructions of the kit. Using the Nanodrop device, the DNA content was measured in the resulting 50 µL extract. For quantitative PCR using an ABI 7900HT sequence detection system (Applied Biosystems, Alcobendas, Spain), DNA was adjusted to 50 ng/2 µL pure water. For amplification, we used the qPCRBIO SyGreen Mix Hi-Rox (PCR Biosystems, London, UK). Each 15 µL SYBR green reaction mixture consisted of 2 µL DNA (50 ng), 7.5 µL SYBR Green PCR-mix (2×), 0.75 µL combined forward and reverse primers (10 µM each), and 4.75 µL distilled water. PCR was performed with 2 min at 95 °C, followed by 40 cycles of 15 s at 95 °C, 30 s at 60 °C, and a separate dissociation step for the melting curve. The specificity of the PCR product was confirmed by ascertaining a single melting peak in the temperature dissociation plots and electrophoresis of the products in an agarose gel (1.5%). All samples were run in duplicate. A positive detection of human DNA in the bmSCs preparation without false positive signals in spinal cord extracts from rats that received no cell treatment was confirmed using primers 5-GGTGAAACCCCGTCTCTACT-3 (forward 101 F) and 5′-GGTTCAAGCGATTCTCCTGC-3′ (reverse 206 R), directed against human Alu elements [31].

### 4.11. Statistical Analysis

In the figure legends, data are presented as the mean values ± standard errors of the mean (SEM). Statistical analysis, performed with GraphPad Prism 8.0 software, consisted of one-factor or two-factor ANOVA, followed by post hoc Dunnett’s comparison tests. Unless stated otherwise, data from animals without SCI were not included in the statistical analysis. In graphical data representation, statistical significance is always indicated as follows: *, #: *p* < 0.05, **, ##: *p* < 0.01, and ***, ###: *p* < 0.001. The asterisks indicate differences from the group without SCI, while the pound symbol is used in comparisons with the SCI group that received control treatment. In the evaluation of tissue loss, results were tested with one-sample *t*-tests.

## 5. Conclusions

The delayed treatment of SCI with fresh non-manipulated human bmSCs provided a limited but substantial functional benefit on sensory–motor recovery in rats. No systemic immune rejection was observed. We conclude that a clinical application for SCI patients in the post-acute or early chronic phase should be tested.

## Data Availability

The datasets generated and analyzed in this study are available from the corresponding author on reasonable request.
